# *npc2*-Deficient Zebrafish Reproduce Neurological and Inflammatory Symptoms of Niemann-Pick Type C Disease

**DOI:** 10.3389/fncel.2021.647860

**Published:** 2021-04-27

**Authors:** Malgorzata Wiweger, Lukasz Majewski, Dobrochna Adamek-Urbanska, Iga Wasilewska, Jacek Kuznicki

**Affiliations:** ^1^Laboratory of Neurodegeneration, International Institute of Molecular and Cell Biology in Warsaw, Warsaw, Poland; ^2^Department of Ichthyology and Biotechnology in Aquaculture, Institute of Animal Sciences, Warsaw University of Life Sciences, Warsaw, Poland

**Keywords:** Niemann-Pick type C, *npc2*, zebrafish model, Nile blue, lysosomal storage disease, neurodegeneration, inflammation, myelin

## Abstract

Niemann-Pick type C (NPC) disease is an autosomal recessive lysosomal storage disease that is caused by a mutation of the *NPC1* or *NPC2* gene, in which un-esterified cholesterol and sphingolipids accumulate mainly in the liver, spleen, and brain. Abnormal lysosomal storage leads to cell damage, neurological problems, and premature death. The time of onset and severity of symptoms of NPC disease are highly variable. The molecular mechanisms that are responsible for NPC disease pathology are far from being understood. The present study generated and characterized a zebrafish mutant that lacks Npc2 protein that may be useful for studies at the organismal, cellular, and molecular levels and both small-scale and high-throughput screens. Using CRISPR/Cas9 technology, we knocked out the zebrafish homolog of *NPC2*. Five-day-old *npc2* mutants were morphologically indistinguishable from wildtype larvae. We found that live *npc2^–/–^* larvae exhibited stronger Nile blue staining. The *npc2^–/–^* larvae exhibited low mobility and a high anxiety-related response. These behavioral changes correlated with downregulation of the *mcu* (mitochondrial calcium uniporter) gene, *ppp3ca* (calcineurin) gene, and genes that are involved in myelination (*mbp* and *mpz*). Histological analysis of adult *npc2^–/–^* zebrafish revealed that pathological changes in the nervous system, kidney, liver, and pancreas correlated with inflammatory responses (i.e., the upregulation of *il1*, *nf*κβ, and *mpeg*; i.e., hallmarks of NPC disease). These findings suggest that the *npc2* mutant zebrafish may be a model of NPC disease.

## Introduction

Cholesterol is a lipid-type organic molecule that is critical for life. It builds, maintains, and modulates cell membranes, where it forms lipid rafts and is involved in endocytosis. It also serves as a precursor of the biosynthesis of essential biomolecules (e.g., vitamin D, sex hormones, and corticosteroids). Alterations of cholesterol metabolism were linked to arteriosclerosis in cardiovascular diseases and neurodegenerative diseases, such as Alzheimer’s disease, and Niemann-Pick type C (NPC) disease.

Niemann-Pick type C disease is a recessive lysosomal storage disease that is caused by a mutation of the intracellular cholesterol transporters *NPC1* (in 95% of cases) or *NPC2* (in 5% of cases), in which un-esterified cholesterol and glycolipids accumulate in lysosomes and late endosomes in the liver, spleen, and brain. The onset of the disease may vary from early childhood (most common) to adulthood, and patients present a wide range of symptoms (Vanier, 2010; Geberhiwot et al., 2018; Seker Yilmaz et al., 2020). Cholestatic jaundice and visceromegaly are often observed as the first signs of this disease. Progressive neurodegeneration is the most devastating and fatal outcome of NPC disease. Neurological symptoms of NPC disease resemble other neurodegenerative disorders, such as Alzheimer’s disease, frontotemporal dementia, and some mitochondrial disorders^1^. In NPC disease, advanced hypotonia, locomotor dysfunction, ataxia, spasticity, dystonia, dysphagia, and dementia, together with developmental delay and regression, often develop in the first decade of life. These changes coincide with a lower volume of the cerebellum, hippocampus, basal ganglia, and thalamus (Geberhiwot et al., 2018). Histological analyses of mouse models of NPC disease identified the loss of Purkinje cells in the cerebellum (Sarna et al., 2003; Ko et al., 2005; Elrick et al., 2010). Aberrant myelination of the central nervous system, coinciding with a decrease in the expression of genes that are associated with oligodendrocyte differentiation (e.g., *Olig1* and *Olig2*), has been observed in *Npc1^–/–^* mice (Yan et al., 2011; Yang et al., 2018). NPC1 disease has other hallmarks, including immune dysfunction (Baudry et al., 2003; Platt et al., 2016), early neuroinflammation, and microglia activation (Cologna et al., 2014; Kavetsky et al., 2019).

The clinical spectrum of NPC disease symptoms ranges from a rapidly progressing prenatal or neonatal form to a chronic form during adolescence. Clinical management guidelines have been developed to help physicians diagnose NPC disease (Geberhiwot et al., 2018). Cholesterol-specific Filipin staining is used as the gold standard for diagnosing NPC disease. Although very useful, this test requires culturing skin fibroblasts, which is rather demanding and cannot be performed in every laboratory. This is why Filipin staining is not recommended as a primary tool for diagnosis. In recent years, several other possible candidate stains and biomarkers have been proposed (Geberhiwot et al., 2018), but there is a need for a more robust method for NPC diagnosis that can be accomplished by genetic analyses.

Although last decade brought progress in the field of NPC, many gaps still need to be filled to improve our understanding of this disease. A further advance in understanding and managing the mechanisms that lead to neurodegeneration that is associated with NPC disease requires the use of high-throughput models, especially based on vertebrates. Zebrafish are a small tropical fish that has been used in laboratories worldwide for more than 30 years. Its significant advantages are genetic similarity to humans (over 70%), its small size, transparent body, and an externally developing body that allows *in vivo* observations at the cellular and organism levels from fertilization until natural death. Their rapid development, high fecundity, and relatively easy husbandry and breeding are other notable advantages. One month of fish life corresponds to approximately 2 years of human life. Most zebrafish studies that are equivalent to the age of patients can be conducted using embryos and early larval stages. The use of zebrafish as a model of human diseases also has economic value. Experiments on embryos are generally inexpensive and fast and can be readily scaled up for high-throughput screening. Zebrafish have been proven to be an excellent model for studies of lipid metabolism (Anderson et al., 2011), neuroinflammation, and neurodegenerative diseases (de Araujo Boleti et al., 2020). A wide range of tools and techniques has been established that allow assessments of somatic and cognitive functions and developmental and pathological processes at both the cellular and organismal levels.

**FIGURE 1:**
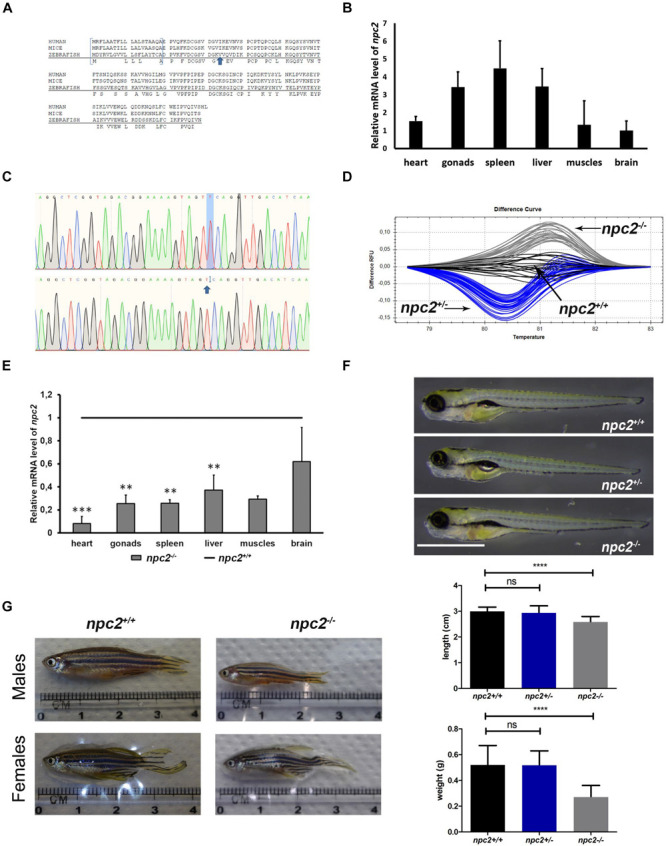
Zebrafish *npc2* mutant. **(A)** Multiple species alignments demonstrated high conservation of the NPC2 protein among vertebrates. Arrows indicate positions of the mutation. **(B)** Expression levels of the *npc2* gene in different tissues in adult fish. Expression was normalized to tissue with the lowest expression (brain). **(C)** Chromatograms confirmed a small deletion in the *npc2* mutant. **(D)** Graphical representation of melt profiles in wildtypes (black), heterozygous mutants (blue), and homozygous mutants (gray) that originated from fish after incrossing *npc2*^±^. **(E)** mRNA levels of the *npc2* gene in various organs in *npc2^–/–^* zebrafish relative to wildtype controls. The data are expressed as the mean ± SEM of three 9-month-old fish per group. Error bars represent the SEM. ***p < 0.001 and **p < 0.01. **(F)** Wildtype, heterozygous, and homozygous *npc2* mutants had indistinguishable phenotypes at 5 dpf. Scale = 1 mm. **(G)** Morphology of adult fish. Smaller body size and weight in 8-month-old *npc2*^–/–^ fish and indistinguishable phenotype of *npc2^±^.* Error bars represent the SD.

The human *NPC1* and *NPC2* genes have homologs in zebrafish. *npc1* mutants were recently created (Lin et al., 2018; Tseng et al., 2018). We applied CRISPR/Cas9 technology and created a zebrafish line with a mutation of the *npc2* gene (Figure 1). The homozygous *npc2* mutant (*npc2^–/–^*) reflects NPC disease, which is a starting point for further studies of NPC disease. We found that Nile blue staining of the external olfactory organ can be used as a robust method for identifying *npc2*^–/–^ larvae, the olfactory organ of which is affected during development.

## Materials and Methods

### Animals

Zebrafish (*Danio rerio*), TL line, were bred and housed at the Zebrafish Core Facility (ZCF) at the International Institute of Molecular and Cell Biology in Warsaw, Poland (license no. PL14656251 from the District Veterinary Inspectorate in Warsaw; license no. 064 and 0051 from the Ministry of Science and Higher Education in Poland). All of the animal procedures were performed in accordance with fundamental ethical principles for the protection of animals that are used for scientific or educational purposes (Act of January 15, 2015; Directive 2010/63/EU). Embryos at 5–13 days postfertilization (dpf) were kept in static tanks with rotifer (*Brachionus plicatilis*) culture (maintained inhouse; rotifer and the rotifer diet were originally purchased from Varicon Aqua Solutions Ltd, United Kingdom). At 2 weeks of age, the fish were kept in circulating water. Fish at 13–30 dpf were fed Gemma Micro three times per day, artemia once a day, and also received rotifers at least once a day. Thereafter, rotifers were excluded while the diet remained unchanged (dry feed three times a day and artemia once a day. The facility maintains a 14 h light/10 h dark photoperiod. The fish were kept in groups of >5 individuals. Larvae and fish up to 6 weeks of age were kept at a maximal density of 50 fish in 3.5 L tanks. From 4 to 6 weeks of age, the fish were kept at a maximal density of 24 fish in 3.5 L tanks. Average values of water quality were the following: salinity of 800 ± 10 μS, pH 7.0 ± 0.2, temperature of 28 ± 0.4°C, undetectable NO_2_^–^ and NH_3_/NH_4_^+^ levels, GH 4, KH 6, dissolved O_2_ of 8.6 ± 0.1 mg/L, and 20% of daily water change. The chosen donor fish were outcrossed to the TL line. Some embryos were sacrificed for genotyping, and the rest were left to grow and used to establish new stable lines with a mutation of the *npc2* gene.

### CRISPR/Cas9

For CRISPR prediction, the following free web tools were used to select target sites: CHOPCHOP^2^, E-CRISP^3^, CRISPRscan^4^, and CCTop^5^. Sequences that were common in at least three predictions and were scored as low risk for off-targets were chosen. The correctness of the sequence in the targeted area in the TL fish at the ZCF was confirmed by Sanger sequencing. Mutagenesis was performed according to the protocol of Gagnon et al. (2014), with modifications (Gagnon et al., 2014). The following gene-specific oligos with T7 overlaps were used: npc2b_(86/34aa) 5′-taatacgactcactataGGTAG ACGGAAAAGTAGTTCgttttagagctagaa-3′ (to create guide IT8). During gRNA preparation, annealing and filling in steps were combined, and the template was prepared for polymerase chain reaction (PCR) using 10 μl of PCR Mix Plus (A&A Biotechnology), 2 μl of gene-specific and constant oligo (5′-AAAAGCACCGACTCGGTGCCACTTTTTCAAGTTGATAA CGGACTAGCCTTATTTTAACTTGCTATTTCTAGCTCTAAA AC-3; each at 100 mM stock), 2 μl dNTPs (100 mM stock), and water up to 20 μl. The PCR conditions were the following: pre-incubation at 95°C for 3 min, followed by 35 cycles of 95°C for 15 s, 40°C for 15 s, and 68°C for 15 s. sgRNA was prepared using 1 μl of gene-cleaned template (145 ng), 0.5 μl T7 RNA polymerase (20 U/μl, A&A Biotechnology), 2 μl NTPs (75 mM each, A&A Biotechnology), 1 μl of buffer (5×, A&A Biotechnology), and water up to 5 μl. The reaction was incubated for 2 h at 37°C. Thereafter, a mixture of 1 μl of RNA-free DNAse (Qiagen) and 14 μl of water was added to each sample. After 15 min of incubation, 10 μl of ammonium acetate (5 M stock) and 60 μl of pure ethanol were added, and the samples were left for precipitation over night at -20°C. gRNA was suspended in water, and the concentration was adjusted to 500 ng/μl. Cas9 protein (14 mg/ml stock, made in-house) was diluted in KCl/HEPES (200 mM/10 mM, pH 7.5) buffer to a final concentration of 600 ng/μl. The injection mixture was assembled fresh by mixing 2 μl of gRNA, 2 μl of gRNA, and 0.4 μl phenol red (Sigma, catalog no. P0290) and left for complex formation at room temperature for 5–10 min. Thereafter, the samples were kept on ice.

### Injections

Microneedles were pulled from borosilicate glass capillaries (Sutter; catalog no. BF 100-50-10) using a P-1000 Flaming/Brown micropipette puller (Sutter) set to the following parameters: heat 542, pull 80, velocity 80, time 170, pressure 500, and RAMP 552. The needle tip was chipped with Dumont no. 5 ceramic-coated fine forceps (Dumostar). One picoliter of the gRNA/Cas9/phenol red mixture was injected into the yolk (just below the zygote) at 1–2 cell-stage zebrafish embryos using a PV 820 Pneumatic PicoPump (World Precision Instruments). Injected embryos were sorted 50/plate and incubated up to 5 dpf in 9 cm diameter Petri dishes that were two-thirds filled with E3 medium in an HPP110 incubator (Memmert) set to 28.5°C, a 14 h/10 h light/dark cycle, 20% light intensity (cold/warm light, 1:1), and 60% humidity.

### DNA Extraction

Fin clipping of adult zebrafish was performed under anesthesia with tricaine (Sigma, catalog no. A-5040). The fish were immersed in 0.7 mM tricaine solution in system water until they stop moving and exhibited no response to tough. Euthanasia of - larvae was performed by an overdose of tricaine. Fish tissue (fragment of the tail fin or pulls of 3–5 dpf embryos) was dehydrated in pure ethanol, incubated at 80°C until complete dryness, soaked in 50–150 μl of TE 10-1 buffer [aqueous solution of 10 mM Tris (pH 8.0) and 1 mM ethylenediaminetetraacetic acid (pH 8.0)] and cooked for 10 min at 95°C. Thereafter, an equal volume of TE 10-1 with 20–60 μg proteinase K (10 mg/ml aqueous stock) was added to each sample. The samples were incubated for 1 h at 55°C. Proteinase K was then inactivated by heat (incubation for 15 min at 95°C). Samples were stored at −20°C before use.

### DNA Sequencing

For sequencing, the amplicon was amplified using PCR Mix Plus (catalog no. 2005–100P, A&A Biotechnology) and npc2_a_F1 5′-GCATATTCGCTGTCATGTGATT-3′ and npc2_b_R1 5′-GTAGGATTGTCCCTTGTGAAGC-3′ primers. The PCR conditions were preincubation at 95°C for 3 min and 35 amplification cycles of 95°C for 15 s, 60°C for 15 s, and 72°C for 15 s, followed by 5 min of incubation at 72°C. The PCR products were purified using EPPiC Fast (catalog no. 1021–100F, A&A Biotechnology) according to the manufacture’s protocol.

### RNA Isolation and cDNA Synthesis

The fish were anesthetized with an overdose of tricaine. RNA was isolated using TRI reagent (Sigma, catalog no. T9424) according to the manufacture’s instructions. The tissue was shredded in 1 ml of solution with a 23-gauge needle. After the addition of 0.2 ml of chloroform, the sample was vortexed and centrifuged at 13,000 rotations per minute (rpm) for 15 min in 4°C. The supernatant was transferred to a new tube, and RNA was precipitated with pure isopropanol overnight at −20°C. After centrifugation at 13,000 rpm for 30 min at 4°C, the pellet was washed with 70% ethanol, air-dried, and resuspended in 15–25 μl of sterile water.

Total RNA was extracted from 5 dpf larvae (*n* = 9/group from at least two independent cohorts), and various organs (brain, liver, heart, spleen, skeletal muscles, and gonads) were collected from 9-month-old male fish (*n* = 4 homozygotes and *n* = 4 wildtypes). Additionally, to test the influence of 2-hydroksypropylo-β-cyclodextrin (2HPβCD) treatment (a drug that is used for the treatment of Niemann-Pick disease) on the expression of selected genes, total RNA was extracted from two groups of treated and untreated zebrafish larvae (5 dpf) from two independent experiments. The RNA template (1,000 ng) was used for cDNA synthesis using the iScript cDNA Synthesis Kit (Bio-Rad).

### High-Resolution Melting (HRM) Analysis

The reaction mixture was composed of (*i*) 5 μl of LightCycler 480 High Resolution Melting Master mix (Roche), 1 μl of MgCl_2_ (25 mM stock), 0.3 μl of each primer (10 μM stock), 0.5 μl of template, and water up to 10 μl or (*ii*) 5 μl of Precision Melt Supermix (Bio-Rad), 0.2 μl of each primer (10 μM stock), 1.0 μl of template, and water up to 10 μl. Two primer pairs were used. npc2a HRM F1 (5′-AACTCTAGTGTGTTGGTTCCTAAC-3′) and npc2a HRM R1 (5′-CAAGTGTACGCGAGAAAAGAAAGTA-3′) were used as controls, which amplified the region upstream of the target site. npc2b HRM F1 (5′-TAATTTCCACTTTCATCTTACAGGC-3′), and npc2b HRM R1 (5′-GGATTGTCCCTTGTGAAGCTTG-3′), which span the mutation site, were used to detect the mutation. HRM analysis was performed on either LightCycler 480 System PCR (Roche) or CFX96 (Bio-Rad), both according to the manufacturer’s protocol. For the Bio-Rad system, the conditions were preincubation at 95°C for 2 min, 35 amplification cycles of 95°C for 10 s and 60°C for 30 s, heteroduplex formation at 95°C for 30 s, followed by 60°C for 1 min, and HRM at 65–95°C with ramp at 0.2°C/10 s. Genotypes were automatically assigned by LightCycler 96 software’s HRM module for the Roche system or Precision Melt Analysis software for the Bio-Rad system and manually annotated as wildtype (black), heterozygous mutant (blue), and homozygous mutant (gray; Figure 1).

### Quantitative PCR Gene Expression Analysis

Gene expression levels were analyzed using the CFX Connect Real-time PCR (RT-PCR) Detection System (Bio-Rad, Hercules, CA, United States). RT-PCR was performed in duplicate using SsoAdvanced Universal SYBR Green Supermix (Bio-Rad, catalog no. 1725274). The data were analyzed using Bio-Rad CFX Maestro 1.0 software. The specificity of the reactions was determined based on dissociation curve analysis. Fold changes were calculated using the ΔΔCq method as described before (Majewski et al., 2019). Expression levels were compared between groups using analysis of variance (ANOVA) followed by Tukey’s Honestly Significant Difference (HSD) *post hoc* test. The 18*S* ribosomal gene was used as a reference. The sets of primers that were used in the analysis are shown in Table 1.

**TABLE 1:** List of primers used for gene expression analysis.

		Primer sequence	
Gene name	Abbreviation	Forward	Reverse
*NPC intracellular cholesterol transporter 2*	*npc2*	5′-aacagggtgtaagaaaaggg-3′	5′-tactttcttttctcgcgtacacttg-3′
*interleukin 1*	*il 1*	5′-tggacttcgcagcacaaaat-3′	5′-gttcacttcacgctcttggatg-3′
*nuclear factor κB subunit 2*	*nfkbeta2*	5′-acatctctgctccatgct-3′	5′-gcagtgaacttgctgaacca-3′
*macrophage expressed 1*	*mpeg*	5′-gtgaaagagggttctgttaca-3′	5′-gccgtaatcaagtacgagtt-3′
*myeloid-specific peroxidase*	*mpx*	5′-gctgctgttgtgctctttca-3′	5′-ttgagtgagcaggtttgtgg-3′
*oligodendrocyte transcription factor 1*	*olig1*	5′-cggactgaaagtttgaagaatgc-3′	5′-tcctgttacccgtaccattcttg-3′
*myelin basic protein a*	*mbpa*	5′-aatcagcaggttcttcggaggaga-3′	5′-aagaaatgcacgacagggttgacg-3′
*myelin protein zero*	*mpz*	5′-cacagcaaaaacagcgtatct-3′	5′-tggggatgggaggctacttt-3′
*myelin proteolipid protein*	*plp1*	5′-acactgttaacgtcctgtcag-3′	5′-ctggtgctttgcatatgttgg-3′
*sry-box transcription factor 10*	*sox10*	5′-aaaacactggggaagctgtg-3′	5′-cgacgtggctggtacttgt-3′
*apolipoprotein Ea*	*apoEa*	5′-tgtggctgtaattgttgcgc-3′	5′-ttccagaactgatccacggc-3′
*calpain 2, (m/II) large subunit a*	*capn2a*	5′-aggctggagaagacatgcac-3′	5′-aggaggaagtttgaagcggg-3′
*claudin 1*	*cldn*	5′-cactgtcactcatcaggtcca-3′	5′-accttcggtacgcaatgtca-3′
*galanin*	*galanin*	5′-atggaccctgaacagtgctg-3′	5′-accaagcagatcttctcgcc-3′
*mitochondrial calcium uniporter*	*mcu*	5′- gtatcccgcattcggtgtct-3′	5′-ctgttctcagaccgtgtgct-3′
*calmodulin-dependent calcineurin a subunit α isoform*	*ppp3ca*	5′-gatgctgcctagcggtgt-3′	5′-agctctcggtggttttgctc-3′
*galectin 3*	*lgals3a*	5′-gaggctttcctgctccaccc-3′	5′- cctcctcctgtgactgcttg-3′
*beclin-1*	*becn1*	5′- ccagctgatggacactgaag-3′	5′- caactccagctgctgtctctt-3′
*autophagy related 5*	*atg5*	5′- cattaaagaggccgatgcac-3′	5′- ctccatgagtttgcgattca-3′
*autophagy related 7*	*atg7*	5′- ctcgaagccttcaaatccac-3′	5′- ggactgaatagcgctccaga-3′
*ubiquitin-binding protein p62*	*p-62*	5′- agcagcctatgggaggattt-3′	5′- cagttgtggaagcaatggag-3′
*18S ribosomal RNA*	*18S*	5′-tcgctagttggcatcgtttatg-3′	5′-cggaggttcgaagacgatca-3′
*elongation factor 1-α*	*EF1α*	5′-cttctcaggctgactgtgc-3′	5′-ccgctagcattaccctcc-3′

### LysoTracker, Filipin, Neutral Red and Nile Blue Staining

Nile blue (B&K, Bytom, Poland) was prepared as a 1% aqueous stock solution. Stock solution of Neutral red (Sigma N7005) was prepared as 2.5 mg/ml solution in water. LysoTracker Red DND-99 (Invitrogen, catalog no. L7528), Neutral red or Nile blue (each at 1:1000 dilution) was added directly into the plates with live 5-day-old zebrafish larvae. After 1 h of incubation at 28°C in the dark, the medium was exchanged to fresh E3 three times over a 15 min period.

The stock of filipin complex (Sigma, catalog no. F9765) was prepared by dissolving 25 mg filipin in 1 ml of dimethylsulfoxide. Aliquoted stocks were stored at −20°C. 5-day-old zebrafish larvae were fixed in 4% paraformaldehyde (Sigma, catalog no. 47608) in 1 × phosphate-buffered saline (PBS; Sigma, catalog no. D5652) for 1 h at room temperature and washed for 5 min three times with 1 × PBS with 1.5% glycine (Sigma, catalog no. G7126). Embryos were stained with Filipin solution (final Filipin concentration of 0.5 mg/ml) in 1 × PBS with 10% bovine calf serum (Sigma, catalog no. 12133C) in the dark for 2 h at room temperature.

Stained larvae were rinsed with 1 × PBS, immersed in 0.7 mM tricaine, transferred to a plate with 3% methylcellulose, and imaged in the ultraviolet channel (Filipin), RFP channel (Lysotracker), and bright field (Nile blue) under a Nikon SMZ25 fluorescent stereomicroscope. The genotype of the imaged fish was confirmed by HRM analysis.

### Treatments

The stock solutions were prepared by dissolving the powdered compound in Milli-Q water to a final concentration of 100 mM for (2-hydroxypropyl)-β-cyclodextrin (2HPβCD; Sigma, catalog no. C0926) and 10 mM for 3-β-[2-(diethylamino)ethoxy]androst-5-en-17-one (U18666A; Sigma, catalog no. 662015). Good-quality eggs were placed in 9 cm diameter Petri dishes filled with 25 ml of E3 medium and kept under static condition at 28 ± 0.5°C. At 3 dpf, the medium was changed to fresh E3 medium supplemented with U18666A (8 μM), 2HPβCD (2 mM), and U18666A (8 μM) together with 2HPβCD (2 mM). As a control, an equal volume of the solvent was used. Embryos were kept in static condition for 2 days. At 5 dpf, the fish were stained with Nile blue and collected for RNA extraction.

### Histology

Adult zebrafish were fixed in neutral buffered 4% formaldehyde solution (Sigma, catalog no. 47608–250ML-F), dehydrated in ethylene, and embedded in paraffin. Longitudinal and transverse 5–6 μm sections were cut using a Leica microtome (RM2265, Leica, Bensheim, Germany) and stained with standard hematoxylin and eosin (H&E). For glycogen and lipofuscin inclusions, periodic acid-Schiff (PAS) reactions were performed according to the manufacturer’s instructions (Sigma-Aldrich, St. Louis, MO, United States). Perl Prussian blue staining to detect hemosiderin was performed according to Orchard (2019). Luxol Fast Blue (LFB), combined with H&E, was used to stain myelin sheaths. The immunohistochemical frequency of CD3 and proliferating cells in the intestinal epithelium and liver was determined in samples that were stained according to the manufacture’s protocol for anti-CD3 (Bond Ready-to-Use Primary Antibody CD3, LN10, Leica Newcastle, United Kingdom) and anti- proliferating cell nuclear antigen (PCNA; 1:400 dilution; clone PC10, DAKO, Poland) antibodies. The colorimetric detection of these cells was performed using DAB (Novolink Polymer Detection Kit, Novocastra, Leica, Newcastle, United Kingdom). The microscopic analysis was performed using NIS Elements software and Nikon Ni-E with NIS Elements software.

### Behavioral Analysis

The behavioral studies of zebrafish larvae were performed as described previously (Wasilewska et al., 2020). Before the experiment, the larvae were kept in a Petri dish (∼50 larvae/dish) that was two-thirds filled with E3 medium in an HPP110 incubator (Memmert) that was set to 28.5°C under a 14 h/10 h light/dark cycle with 20% light intensity (cold/warm light, 1:1) and 60% humidity. The larvae (5 dpf) were transferred to 12-well plates and placed in a ZebraBox high-throughput monitoring system (ViewPoint Life Sciences, Lyon, France). The experiments were performed in a volume of 2 ml of E3 medium, and the light intensity was set to 70%. Locomotor activity was recorded for 15 min using ZebraBox. A total of 40 wildtype and 32 *npc2^–/–^* larvae from two independent cohorts were used in the experiment.

The video files were further analyzed using EthoVision XT software (Noldus, Wageningen, Netherlands). The data were exported to Microsoft Excel files and further analyzed using Excel (Microsoft, Redmond, WA, United States) and R software (R Foundation for Statistical Computing, Vienna, Austria, R package version 3.6.2). The experiment was divided into three 5 min periods, and the mean total distance traveled (mm), mean velocity (mm/s), and mean movement duration were calculated and compared independently for these time bins. The Wilcoxon rank-sum test was used for comparisons between wildtype and *npc2^–/–^* larvae.

Additionally, the area of the well was divided into borders and a central area. This allowed analyses of the level of thigmotaxis that was calculated according to the following formula: [duration of movement (in borders or center) + duration of no movement (in borders or center)]/[duration of movement (total) + duration of no movement (total)] × 100% (Wasilewska et al., 2020). The data are expressed as medians with first and third quartiles using boxplots, and dots represent data outliers. Heatmaps that represent average traces of wildtype and *npc2^–/–^* larvae were generated using EthoVision XT software.

### Statistical Analysis

In quantitative PCR gene expression analysis, expression levels were compared between groups using ANOVA followed by Tukey’s HSD *post hoc* test. In the behavioral studies the Wilcoxon rank-sum test was used for comparisons between wildtype and *npc2^–/–^* larvae.

## Results

### Generation and Phenotype of the *npc2* Zebrafish Mutant

The zebrafish *npc2* gene (ENSDARG00000090912) maps to chromosome 17 and encodes a 149 amino acid protein that shares over 64% sequence identity with its human ortholog (Figure 1A). Expression of the *npc2* gene could be found in each tested tissue (i.e., brain, heart, liver, muscles, skin, and reproductive organs; Figure 1B). Using CRISPR/Cas9 technology and gRNAs that targeted the *npc2* gene, we generated F0 carriers of different mutations. After an initial screening by HRM analysis, six donor fish with the most distinct HRM patterns were bred, and their F2 offspring were analyzed by HRM and sequencing. A mutant line with a 1 bp deletion in the third exon of the *npc2* gene (ENSDARG00000090912) that is predicted to introduce a premature stop codon was selected for further studies (Figure 1C). The deletion caused a characteristic shift in the melting temperature of the amplicon that was identified by HRM (Figure 1D). In the mutant, qPCR analysis confirmed a significantly lower level of *npc2* transcripts (Figure 1E). At 5 dpf, the morphology and length of the hetero- and homozygous*npc2* mutants were indistinguishable from wildtype siblings (Figure 1F), similar to *npc1* zebrafish knockouts (Lin et al., 2018; Tseng et al., 2018). Over time, the differences became more apparent. The 2-month-old *npc2^–/–^* fish were smaller than their siblings, and the sex ratio of homozygotes was skewed toward males (data not shown). When separated from their siblings, the *npc2^–/–^* fish did not need to compete for food and grew faster. From 4 months of age, their motor functions were clearly impaired (Supplementary Video 1) as compared to wildtype siblings (Supplementary Video 2). The malnutrition and progressive loss of motor skills continued to worsen, but not all *npc2^–/–^* fish were equally affected. Some fish had to be euthanized at the age of 5 months because their eating and swimming skills were severely impaired, whereas others were fit and survived four more months. The length and weight of the 9-month-old *npc2^–/–^* fish were significantly reduced (Figure 1G). The *npc2^–/–^* fish bred normally, whereas inbreeding of the *npc2^–/–^* fish was unsuccessful (data not shown). Interestingly, we could obtain offspring by crossing *npc2^–/–^* males with either wildtype or *npc2*^±^ females.

### Nile Blue Staining Identifies *npc2^–/–^* Larvae

Although, nowadays not recommended as a primary tool, the cholesterol-specific filipin staining for many years was used as a gold standard in NPC diagnostics (Geberhiwot et al., 2018). That is why we applied this stain to the mutant fish and confirmed the presence of blighter signals in the *npc2^–/–^* mutant (Supplementary Figure 1). This finding indicates that as expected, un-esterified cholesterol accumulates in homozygous *npc2* mutant.

Niemann-Pick type C disease is a lysosomal storage disease. We used the LysoTracker red probe to visualize acidic organelles *in vivo* to quickly identify the mutant. We observed increases in LysoTracker red staining in neuromasts in some *npc2^–/–^* larvae (Supplementary Figure 2). However, using this staining, we failed to reliably distinguish wildtype from *npc2^–/–^*.

**FIGURE 2:**
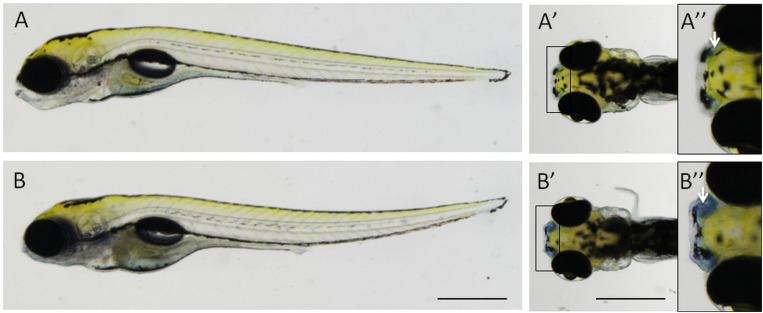
Nile blue staining distinguishes *npc2^–/–^* fish from wildtype and heterozygous siblings. The strong signal could be detected in the peripheral olfactory organ in 5-day-old *npc2^–/–^* larvae **(B–B”)**, unlike their siblings **(A–A”)**. **(A,B)** lateral view; **(A’,A”,B’,B”)** dorsal view; and **(A”,B”)** enlarged fragments of **A’,B’**, respectively. Arrows indicate the position of the olfactory organ. Scale bar = 0.5 mm.

We found that Nile blue staining allowed the robust and reliable detection of *npc2^–/–^* larvae. When added to the water in the dish, Nile blue reversibly stained live larvae in a few minutes. At 5 dpf, strong staining in the peripheral olfactory organ was specific (certainty > 95%) to *npc2^–/–^* fish, whereas staining in other parts of the body appeared random (Figure 2).

**FIGURE 3:**
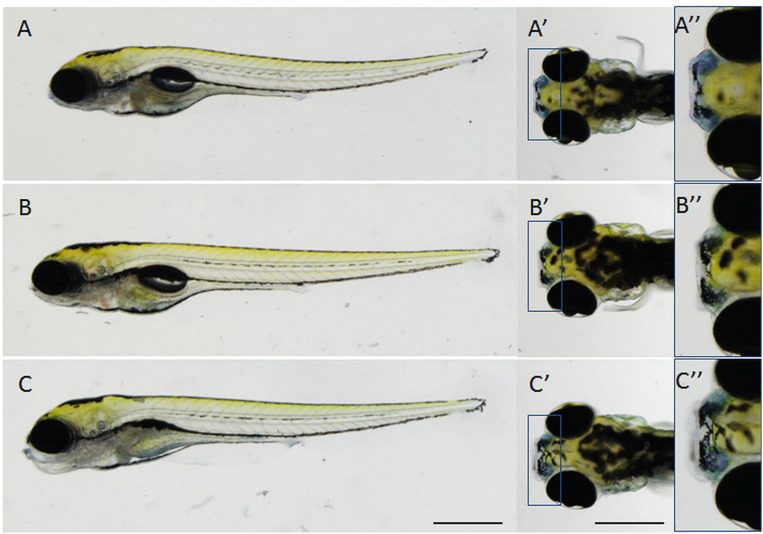
Specificity of Nile blue staining in the olfactory organ in *npc2^–/–^* larvae at 5 dpf. **(A–A”)** untreated *npc2^–/–^* larvae; **(B–B”)** treatment with 2 mM 2-hydroxypropyl-β-cyclodextrin (2HPβCD; a drug that is used in clinical trials to treat NPC patients) decreased staining intensity to wildtype levels (Figures 2A–A”); and **(C–C”)** treatment with 8 mM (3β)-3-(2-[diethylamino]ethoxy)androst-5-en-17-one hydrochloride (U18666A, an inhibitor of cholesterol synthesis that is used to model NPC chemically) increased staining intensity in *npc2^–/–^* larvae. **(A–C)** lateral view; **(A’,A”,B’,B”,C’,C”)**, dorsal view; and **(A”,B”,C”)** enlarged fragments of **A’,B’,C’**, respectively. Scale bar = 0.5 mm.

Nile blue stains lipids. It has been primarily used to detect melanin and lipofuscin (Lillie, 1958). Nile blue-based dyes were later discovered to localize in lysosomes. Their lysosomotropic photosensitizer properties could be used to target and kill tumor cells (Gattuso et al., 2016; Martinez and Henary, 2016). To verify the specificity of Nile blue staining, we applied it to larvae that were treated with 2HPβCD (a drug that is used in clinical trials to treat NPC patients) and U18666A (an inhibitor of cholesterol synthesis that is used to chemically model NPC disease). 2HPβCD treatment reduced the intensity of blue staining in the nose in *npc2^–/–^* fish, whereas U18666A treatment increased this signal (Figure 3). The intensity of staining in wildtype fish and heterozygous *npc2* mutant fish followed the same trend, in which staining was weaker in 2HPβCD-treated larvae and stronger in U18666A-treated larvae (data not shown).

### Decrease in Locomotor Activity in *npc2*^–/–^ Zebrafish Larvae

**FIGURE 4:**
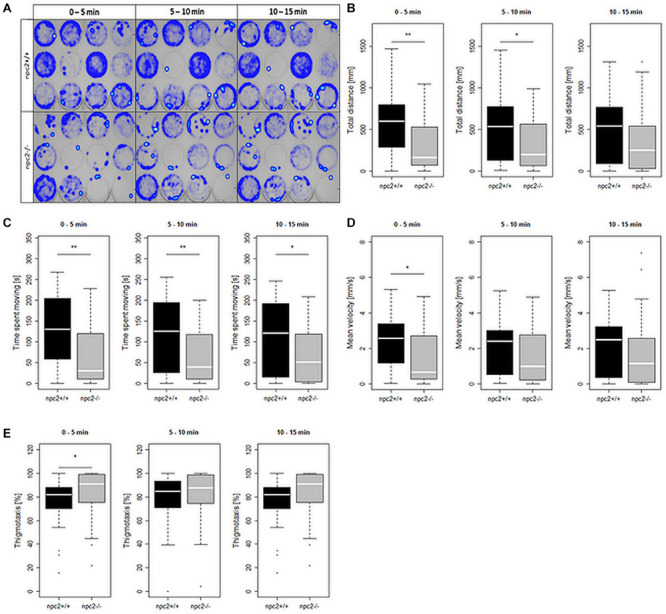
Decrease in mobility and increase in thigmotaxis in *npc2*^–/–^ larvae. **(A)** Heatmaps of mean traces of *npc2*^+/+^ and *npc2*^–/–^ larvae during each 5 min period. **(B–E)** Boxplots of total distance traveled, time spent moving, mean velocity, and thigmotaxis in *npc2*^+/+^ and *npc2*^–/–^ larvae. **(E)** *p < 0.05 and **p < 0.01.

To investigate the effect of *npc2* mutant on behavior, locomotor activity in *npc2*^+/+^ and *npc2*^–/–^ zebrafish larvae was analyzed. A significant reduction of mobility was observed in *npc2^–/–^* larvae compared with their wildtype siblings (Figure 4A). Mutants traveled a shorter distance (Figure 4B), and they remained mobile for a shorter period of time (Figure 4C). The mean velocity during the first 5 min of the experiment was lower in *npc2^–/–^* larvae compared with *npc2*^+/+^ larvae (Figure 4D). During the initial 5 min of the experiment, mutant larvae exhibited a stronger tendency to stay in close proximity to the borders of the well (thigmotaxis; Figure 4E). This phenomenon is a typical anxiety-like response in zebrafish larvae (Lundegaard et al., 2015), suggesting a stronger anxiety-related response to a stressor in this group.

### Pathological Changes in Internal Organs in Adult *npc2*^–/–^ Fish

**FIGURE 5:**
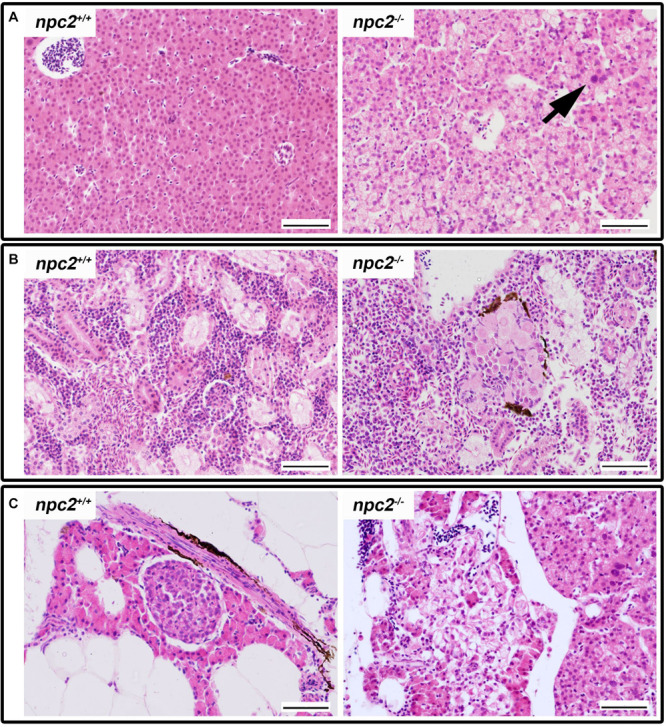
Pathological changes in soft tissues in *npc2^–/–^* revealed by H&E staining. **(A)** Cells with enlarged nuclei (black arrow), hepatosteatosis with different type of fatty droplet accumulation (open arrowhead) and foam cytoplasm (asterix) were found in *npc2*-deficient livers. **(B)** Kidneys with degenerating cells (DMc) between ductal (DT) and proximal (PT) tubules. **(C)** Pancreas with degeneration of zymogen granules in acinar cells and cholesterol deposits. The paraffin sections are from adult *npc2^–/–^* fish and wildtype *npc2*^+/+^ control. Scale bar = 50 μm.

The histopathological analysis of adult *npc2*^–/–^ larvae revealed lesions in the spleen, liver, and pancreas. In the liver in *npc2^–/–^* fish, numerous foam hepatocytes, hepatocytes that contained fat droplets, and cells with enlarged nuclei were observed (Figure 5A). An atypical interstitial group of foam cells was observed in the anterior part of the *npc2^–/–^* kidney (Figure 5B). The structure of the pancreas was disturbed. Acinar cells in the pancreas in *npc2^–/–^* larvae had no characteristic zymogen granules but had multiple circular cholesterol inclusions that were not observed in the control group (Figure 5C).

**FIGURE 6:**
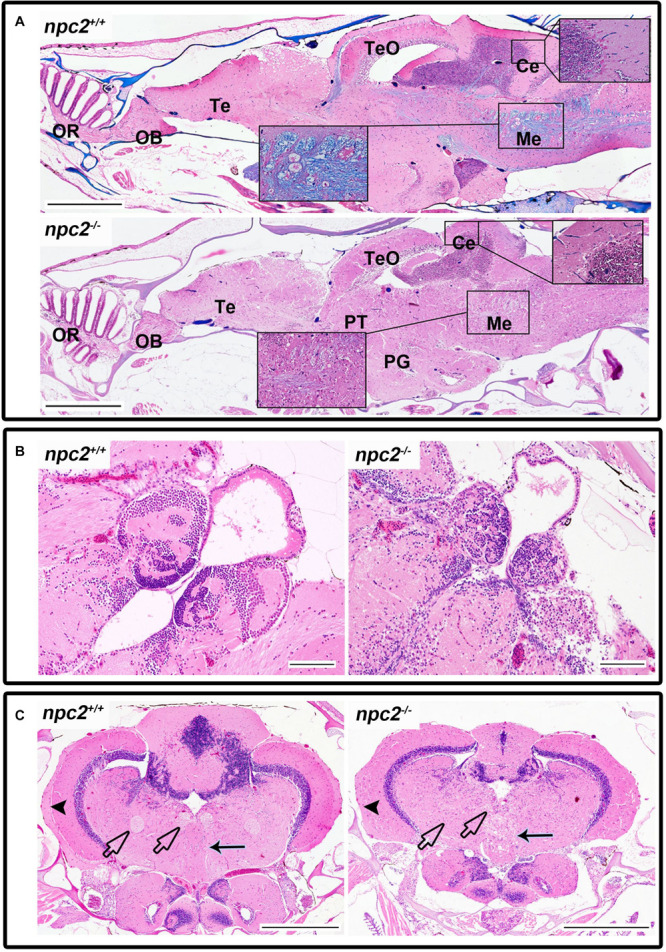
Pathological changes in the central nervous system in adult *npc2^–/–^* zebrafish. **(A)** Luxol fast blue staining showed differences in myelination between wildtype and *npc2^–/–^* fish. **(B)** Changes in habenula structure were present in some *npc2^–/–^* fish, which lacked the characteristic structure of this part of the habenular tract. **(C)** Midbrain with vacuolization of the habenula tract (blue arrow) with fascicular retroflexus (blue arrowhead) and degenerative changes in the medial and lateral longitudinal fascicle. OB, olfactory bulb; OR, olfactory rosette; Te, telencephalon; TeO, tectum opticum; Ce, cerebellum; Me, medulla; PG, preglomerular complex; and PT, posterior tuberculum. **(A)** LFB staining. Scale bar = 500 μm. **(B,C)** H&E staining. Scale bar = 100 μm. **(C)** scale bar = 500 μm.

**FIGURE 7:**
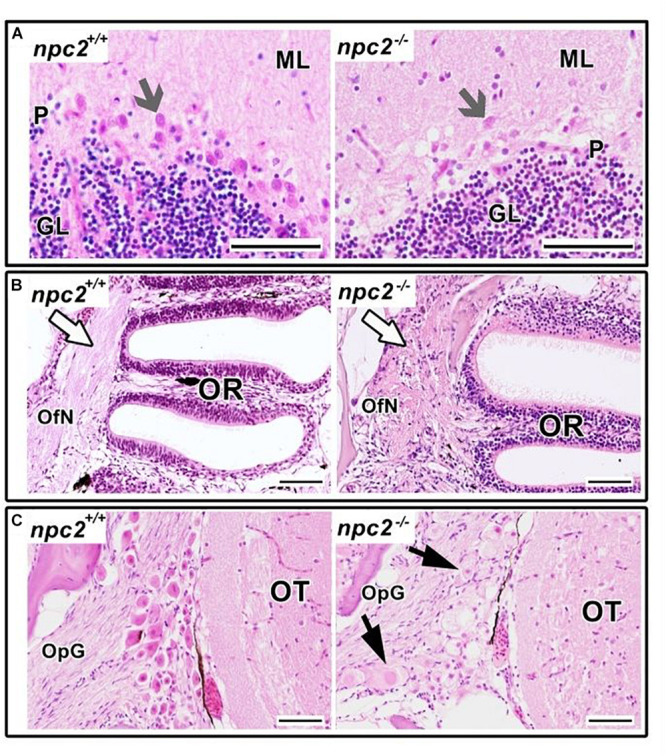
Pathological changes in *npc2*^–/–^ sensory organs. **(A)** Cerebellum structure with Purkinje cells degeneration in *npc2* mutant (gray arrow), **(B)** Olfactory rosette with olfactory nerves (white arrow). **(C)** Optic nerve ganglion. Cells with a foamy, pale cytoplasm, eosinophilic nuclei, and signs of degeneration (black arrows) within the optic tectum. **(A)** ML, molecular layer; P, Purkinje cell leyer; GL, granule cell layer; OfN, olfactory nerves; and OR, olfactory rosette; **(B)** OT, optic tectum; OpG, optic nerve ganglion. H&E staining. Scale bar = 50 μm.

Morphological and histopathological changes were also observed in the brain in *npc2*^–/–^ fish (Supplementary Figure 3 and Figure 6). The myelination of axons in *npc2^–/–^* fish was affected (Figure 6A), especially in the habenular commissure (Figures 6B,C). The habenula’s characteristic layered structure was affected. The fascicular retroflexus, interpeduncular nucleus, and raphe were vacuolated, and the myelination of axonal tracts decreased (Figures 6B,C). The two mechanoreceptive areas with well-differentiated axonal tracts (lateral and medial longitudinal fascicle) were observed only in wildtype fish compared with *npc2^–/–^* fish (Figure 6C). In the cerebellum, Purkinje cells were more diffuse than in the control group, and the structure on these cells differed from control fish (Figures 6C, 7A). Purkinje cells had spheroid, highly non-condensed chromatin, enlarged nuclei, and a distended perikaryon (Figure 7A). In this region, no PCNA-positive cells were observed, although proliferation was detected in the parvocellular preoptic nucleus and caudal zone of the periventricular hypothalamus compared with the control group (Supplementary Figure 4A).

In the olfactory sensory epithelium, no histopathological changes were observed, although the olfactory nerve had some degenerative attributes, characterized by a shrunken cytoplasm of axons that were visible in transversal sections. Similar histopathological changes were observed in the telencephalon and rhombencephalon (Figure 6A). In the external and internal cellular layers of the olfactory bulb, multiple vacuolations were observed in intracellular matter, but no neural cytopathological changes were found. Widespread foamy vacuolation of the cytoplasm of neurons and glia and neuronal spheroids were present in the *npc2*^–/–^ telencephalon and optic tectum (mostly in the stratum fibroetgricialem). Similar changes were observed in the olfactory bulb and optic nerve (Figures 7B,C). CD3-positive cells were observed in *npc2^–/–^* fish, whereas no signal was detected in the wildtype group (Supplementary Figure 4B).

**FIGURE 8:**
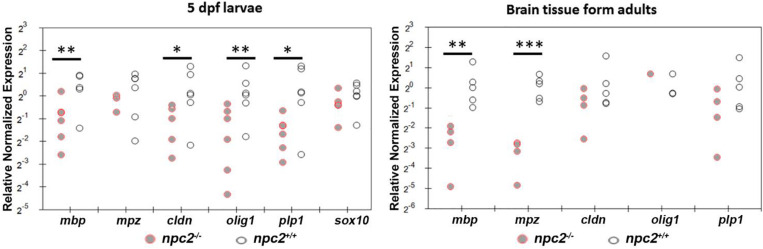
Decrease in myelination in *npc2*-deficient larvae and adult fish. Scatter plots show the normalized expression of selected genes in 5 dpf larvae and in the brain in *npc^–/–^* and wildtype zebrafish. Each circle corresponds to one zebrafish. The 18*S* ribosomal gene was used as a reference. At least three samples were analyzed. ***p < 0.001; **p < 0.01; and *p < 0.05.

We determined the level of gene expression in wildtype and *npc2^–/–^* fish at two developmental stages. Homozygous larvae were preselected based on Nile blue staining. The genotype of both 5-day-old larvae and adult fish was confirmed by HRM analysis. In whole larvae, the *mbp, cldn*, and *olig*1 genes were down-regulated, whereas expression of the *mpz*, *plp1*, and *sox10* genes was unchanged. In the *npc2^–/–^* adult brain, a significant reduction of expression was detected only for the *mbp* and *mpz* genes (Figure 8). The downregulation of myelination markers was consistent with the decrease in LFB staining (Figure 6A).

### Alterations of Expression of Genes That Shape Inflammation, Autophagy and Ca^2+^ Signaling in *npc2^–/–^* Fish

**FIGURE 9:**
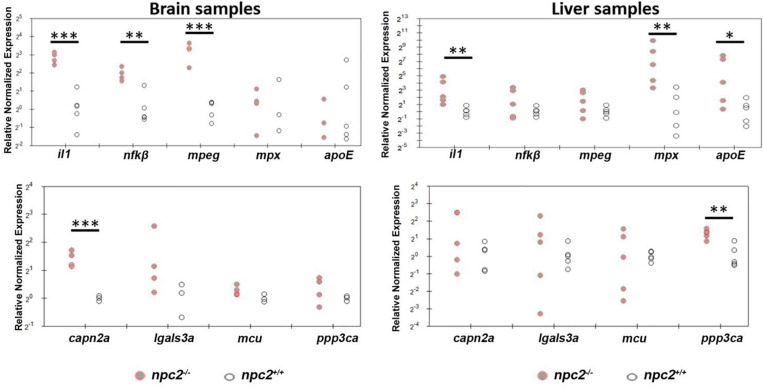
Signs of inflammation and alterations of Ca^2+^ homeostasis in *npc2^–/–^* zebrafish. Scatterplots show the normalized expression of selected genes in brain and liver tissue from 9-month-old *npc2^–/–^* homozygotes and wildtype zebrafish. Each circle corresponds to one zebrafish. The 18*S* ribosomal gene was used as a reference. Samples from at least three fish were analyzed. ***p < 0.001, **p < 0.01, and *p < 0.05.

The downregulation of *mbp* expression is associated with the upregulation of interleukin-1 signaling in *npc1^–/–^* animal models (Mannie et al., 1987; Qiao et al., 2018). To determine whether *npc2^–/–^* zebrafish exhibit hallmarks of inflammatory responses that are associated with NPC disease, we profiled the expression of selected genes in the brain and liver in 9-month-old fish. In the brain, the upregulation of *il1*, *nf*κβ, *mpeg*, and *capn2a* was detected (Figure 9). In the liver, *il1* expression but not *nf*κβ, *mpeg*, or *capn2a* expression was also up-regulated. However, in contrast to the brain, *mpx*, *apoE*, and *ppp3ca* expression was up-regulated (Figure 9).

In addition, we stained zebrafish larvae with Neutral red, a dye which specifically labels microglia in the larval zebrafish brain. In 5 dpf *npc2^–/–^* larvae, we found numerous red-labeled cells with dark red aggregates having typical appearance of over-activated micro-microglia, i.e., soma size was enlarged and cells had amoeboid shape (Supplementary Figure 5). Beclin-1 plays an important role autophagy during neurodegeneration. We found that *npc2^–/–^* larvae show slightly increased expression level of *becn1*, which is in line with previous finding of Pacheco and colleagues who demonstrated that enhanced basal autophagy in NPC1 deficiency is mediated by increased expression of *Beclin-1* (Pacheco et al., 2007). We did not detect significant changes in the expression level of markers of ubiquitin-dependent stress-induced autophagy (*atg5*, *atg7*, and *p62*; Supplementary Figure 6).

**FIGURE 10:**
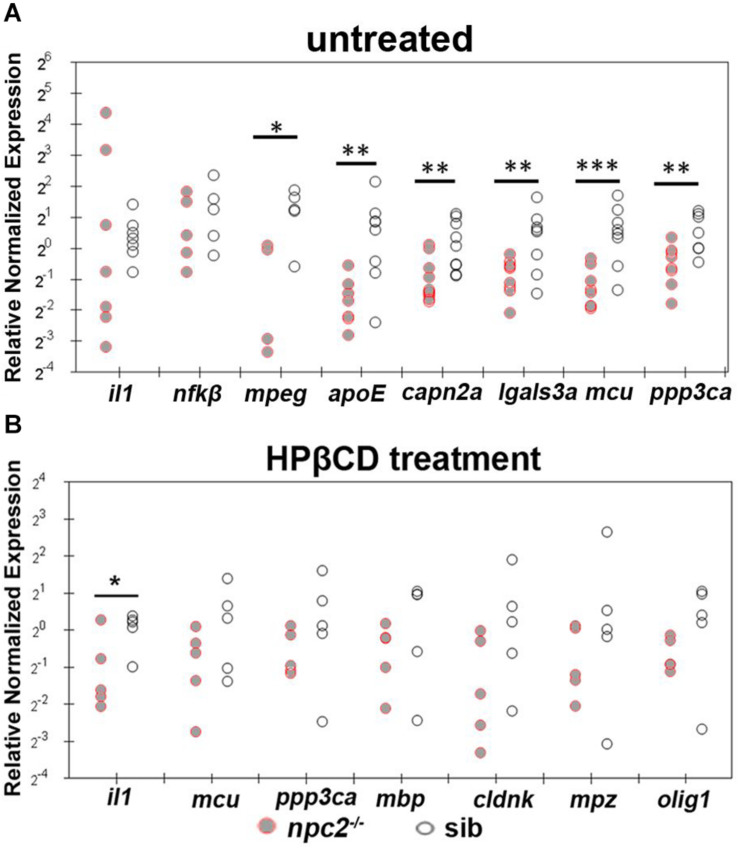
Treatment with 2-hydroxypropyl-β-cyclodextrin increased the expression of genes that are related to inflammation and Ca^2+^ homeostasis in 5 dpf *npc2^–/–^* larvae to wildtype levels. Scatterplots show the normalized expression of selected genes in untreated larvae **(A)** and larvae that were treated with 2 mM 2HPβCD **(B)** at 5 dpf. Each circle corresponds to one zebrafish. The 18*S* ribosomal gene was used as a reference. The experiments were performed in triplicate. ***p < 0.001, **p < 0.01, *p < 0.05.

We also found that *apoE*, *capn2a*, *lgals3a*, *mcu*, *mpeg*, and *ppp3ca* expression were down-regulated in 5 dpf *npc2^–/–^* larvae compared with wildtype fish (Figure 10A). The most striking difference was related to the level of *mcu*, with very high statistical significance. These changes appeared to be specific to NPC disease because 2HPβCD treatment rescued the levels of *mcu* and *ppp3ca* expression and the expression of genes that are associated with myelination (*olig1, mbp, cldn*, and *plp1*; Figures 8, 10B).

## Discussion

Niemann-Pick type C disease is a rare disease. The elucidation of its pathogenesis can help understand the molecular mechanisms that underlie more common disorders that are related to impairments in cholesterol homeostasis, autophagy, inflammation, and neurodegeneration. Accumulating evidence links cholesterol transport inhibition with lower susceptibility to viral infection, including SARS-CoV-2 (Gong et al., 2016; Wec et al., 2016; Stoeck et al., 2018; Ballout et al., 2020; Sturley et al., 2020; Vial et al., 2020), and studies of NPC genes are highly relevant. Of two human NPC genes, NPC1 and its homologs have been the most studied (Fog and Kirkegaard, 2019; Wheeler and Sillence, 2020). Various tools have been created and successfully applied to advance our knowledge of NPC1 function in normal and pathological processes (Lin et al., 2018; Tseng et al., 2018; Fog and Kirkegaard, 2019). Surprisingly, little is known about NPC2, a soluble lysosomal protein that delivers cholesterol to NPC1 (Deffieu and Pfeffer, 2011). Homologs of both the human *NPC1* and *NPC2* genes are conserved in zebrafish. Zebrafish *npc1* mutants were recently described (Lin et al., 2018; Tseng et al., 2018). To our knowledge, no zebrafish mutant is available for *npc2*. To fill this gap in the literature, we created and characterized homozygous *npc2^–/–^* fish.

Clinical manifestations in humans who are deficient in NPC1 or NPC2 are similar. Hence, unsurprising is that *npc2*^–/–^ zebrafish in many ways resemble the phenotype of *npc1^–/–^* fish. In the present study, we found that the manifestation of the *npc2* mutation resembles changes that are seen in human patients. NPC disease can be highly heterogeneous in patients (Vanier, 2010; Reunert et al., 2016). In *npc2* mutant fish, we observed variability in symptoms and their intensity. Similar to patients, *npc2^–/–^* fish exhibited clear signs of neuropathy and cytological changes in the liver and pancreas. Fish do not have bone marrow, and hematopoietic processes are conducted by the thymus, spleen, and anterior kidney. These three major hematopoietic organs were affected, and cells with a characteristic foam cytoplasm were observed, similar to several human diseases (Eom et al., 2015) and animal models (Kuemmel et al., 1997) of renal degeneration. Foam cells within the *npc2^–/–^* brain were also present in NPC patients and mouse models. Defects in Purkinje cells in the cerebellum were found in adult *npc2^–/–^* fish. In addition to histological changes, *npc2^–/–^* fish also recapitulated molecular changes, including alterations of the expression of genes that are involved in myelination and inflammation. However, gene expression was not changed in the same manner in larvae and adults. This is unsurprising because adult fish were severely affected, whereas the disease just started to progress in 5-day-old larvae.

Links between mitochondria, lysosomes, and neurodegeneration have been confirmed previously (Saffari et al., 2017; Torres et al., 2017). Mitochondrial dysfunctions have also been described in human cells with a mutation the human *NPC1* gene and in corresponding mouse models (Woś et al., 2016; Erickson et al., 2020). We found a significant decrease in expression of the *mcu* gene in *npc2^–/–^* larvae. 2HPβCD treatment rescued expression levels. To our knowledge, this is the first study that found that impairments in calcium uptake into mitochondria may contribute to NPC disease. We previously reported that *mcu* knockout can rescue *pink1* mutant zebrafish, a model of Parkinson’s disease, from the loss of dopaminergic neurons (Soman et al., 2019). Alterations of calcium homeostasis and signaling are not restricted to mitochondria. NPC disease was linked to a decrease in the expression of *Ppp3ca* in the mouse cerebellum (Reddy et al., 2006). Significant reductions of *ppp3ca* expression were also observed in *npc2^–/–^* larvae, whereas expression in the adult mutant brain appeared to be unaffected. We also found an increase in *ppp3ca* expression in the *npc2^–/–^* liver. The regulation of expression levels may be tissue-dependent and vary within more complex structures. In such a case, the analysis of the whole brain could have masked changes that occurred in the cerebellum.

In humans and zebrafish, the loss of one allele of the *NPC1* or *NPC2* gene is insufficient to cause NPC disease. The homozygous mutation of either *npc* gene leads to premature death. Some individuals can live longer, whereas some die at a young age. The prevalence of the early infantile form of NPC disease is much higher in children with a mutation of the *NPC1* gene compared with patients with the mutation of *NPC2* (Seker Yilmaz et al., 2020). The majority of *npc2^–/–^* fish live longer and can reach the age of 4 months, whereas the majority of *npc1^–/–^* fish die within the first month of life. Assuming that 1 month of zebrafish life corresponds to 2 years of human life, the lifespan of patients and *npc2^–/–^* zebrafish is similarly affected.

A link between Niemann-Pick disease and growth restriction has been established for Niemann-Pick type B disease, which is a lysosomal storage disease that is associated with the accumulation of sphingolipids (Wasserstein et al., 2003). This link has not yet been established for NPC disease. Homozygous mutations of *npc1* (Lin et al., 2018; Tseng et al., 2018) and *npc2* (present study) in zebrafish result in significant reductions of body length and weight compared with wildtype and heterozygous siblings. Tseng et al. suggested that “the growth defect observed in the *npc1* mutant zebrafish may be due to impaired feeding” (Tseng et al., 2018). Lawrence et al. reported that the sex ratio in zebrafish is strongly influenced by the feeding regimen, and fish that eat more are more likely to become female (Lawrence et al., 2008). *npc2^–/–^* fish and their heterozygous and wildtype siblings occupied the same tank. The sex ratio is skewed in *npc2* mutants, and *npc2^–/–^* fish tend to be males, whereas their wildtype siblings tend to be mostly females. Thus, growth restriction and male-rich population of *npc2^–/–^* fish may be at least partially attributable to an inability to take food. Moreover, anatomical and histological changes in the olfactory and optic neuron tracts and degenerative changes in mechanosensory fibers may hinder the ability of *npc2^–/–^* fish to find food.

Despite the smaller body size, adult *npc2^–/–^* fish produced gametes, but our attempts to inbreed these fish were unsuccessful. A similar problem was reported for *Npc2^–/–^* mice (Busso et al., 2010, 2014) and *npc1^–/–^* zebrafish (Tseng et al., 2018). However, we found that male *npc2^–/–^* fish could occasionally mate with *npc2*^±^ female fish, resulting in viable offspring. This finding is consistent with the observation by Busso et al. (2014) that male *Npc2^–/–^* mice generated sperm that could fertilize eggs *in vitro*, although they did not mate. These authors suggested that locomotor dysfunction prevents male mice from breeding, but other factors might contribute to this problem. Abnormal cerebellar morphogenesis could be one of the factors (Caporali et al., 2016). Mating behavior involves social integrations, in which odor communication plays an important role. Impairments in the sense of smell is an early marker of neurodegenerative diseases. We found pathological changes in the olfactory system in *npc2^–/–^* fish. Sensory deficits that are associated with pronounced neurodegeneration in the olfactory system have also been described in *Npc1^–/–^* mice (Hovakimyan et al., 2013). Therefore, tremor, defective movements, and an inability to respond to chemical cues likely prevented animals with NPC disease from successfully breeding.

Niemann-Pick type C disease patients often exhibit neuropsychiatric symptoms (Evans and Hendriksz, 2017; Mengel et al., 2017; Patterson et al., 2017). In behavioral tests, *npc2^–/–^* larvae exhibited a range of behavioral changes, including an increase in anxiety-like behavior upon stress exposure. Studies showing decreased exploratory activity, hyperactivity and reduced anxiety were performed on mice, but only on adult animals which motor skills already were impaired and hence their performance may have been affected (Võikar et al., 2002; Gómez-Grau et al., 2017). Similar to fish, behavioral changes can be quantified in 5-day-old tremor-free animals, and disease progression can be followed over time. The test may be scalable. The zebrafish model of NPC disease presents unique features that may help efficiently and reliably detect early changes, monitor disease progression, and assay drug efficiency.

Although several NPC models exist, many gaps still need to be filled to improve our understanding of this disease. The *npc2* mutant makes an important contribution to the range of existing models that allow further studies of NPC disease and other neurodegenerative cholesterol-dependent disorders at the organismal, cellular, and molecular levels. Experiments in zebrafish are scalable and suited for high-throughput drug screens. The zebrafish *npc2* mutant may be utilized as a helpful model for the drug development pipeline in early stages of NPC disease. Another issue is the clinical diagnostic value of Nile blue, a dye that specifically provides a strong stain in the nose in *npc2^–/–^* larvae.

## Data Availability Statement

The raw data supporting the conclusions of this article will be made available by the authors, without undue reservation.

## Ethics Statement

All activities were performed in compliance with fundamental ethical principles (Protection of animals used for scientific or educational purposes, Act of January 15, 2015; Directive 2010/63/EU). The majority of the experiments were performed in 5-day-old zebrafish larvae (a stage at which zebrafish are not considered protected animals). The experiments did not qualify as a subject of review by the local ethical committee. In the case of adult fish, recording their natural behavior was performed without any interference. The fish remained in their home tank, and no handling or any other intervention that could cause any pain or distress was applied to them. Consequently, this action did not classify as a procedure and did not require any permit from the ethics committee.

## Author Contributions

MW, LM, IW, and JK conceived and planned the experiments. MW, LM, IW, and DA-U performed the experiments. MW, LM, IW, and DA-U contributed to sample preparation. MW, LM, IW, DA-U, and JK contributed to interpretation of the results. MW took the lead in writing the manuscript. All authors provided critical feedback and helped shape the research, analysis, and manuscript.

## Conflict of Interest

The authors declare that the research was conducted in the absence of any commercial or financial relationships that could be construed as a potential conflict of interest.

## Footnotes

1 https://rarediseases.org/rare-diseases/niemann-pick-disease-type-c/

2 http://chopchop.cbu.uib.no/

3 http://www.e-crisp.org/E-CRISP/

4 http://www.crisprscan.org/?page=gene

5 https://crispr.cos.uni-heidelberg.de/index.html

## Supplementary Material

Supplementary Image 1 Un-esterified cholesterol accumulation in the *npc2* mutant.

Supplementary Image 2 LysoTracker red staining of the *npc2* mutant.

Supplementary Image 3 Morphology of the *npc2*^–/–^ brain.

Supplementary Image 4 Immunohistochemical localization of proliferating nuclear antigen and CD3 in central nervous system tissue in *npc2*^–/–^ fish. A.

Supplementary Image 5 Neutral red staining of the *npc2* mutant at 5 dpf.

Supplementary Image 6 Expression of the autophagy markers in *npc2*-deficient larvae and adult fish.

Supplementary Video 1 Natural behavior in adult *npc2*^–/–^ fish differs from wildtype controls. Similar to human patients, locomotor functions were impaired in *npc2*-deficient fish, reflected by uncoordinated swimming.

Supplementary Video 2 Natural behavior in adult wildtype fish.
