# Supplementary material for: npc2-Deficient Zebrafish Reproduce Neurological and Inflammatory Symptoms of Niemann-Pick Type C Disease
Source: Front Cell Neurosci. 2021 Apr 27;15:647860. doi: 10.3389/fncel.2021.647860 (PMC8111220; doi:10.3389/fncel.2021.647860)
Supplement: Supplementary Image 1 — Un-esterified cholesterol accumulation in the npc2 mutant. [file Image_1.pdf]

# *Supplementary Material*

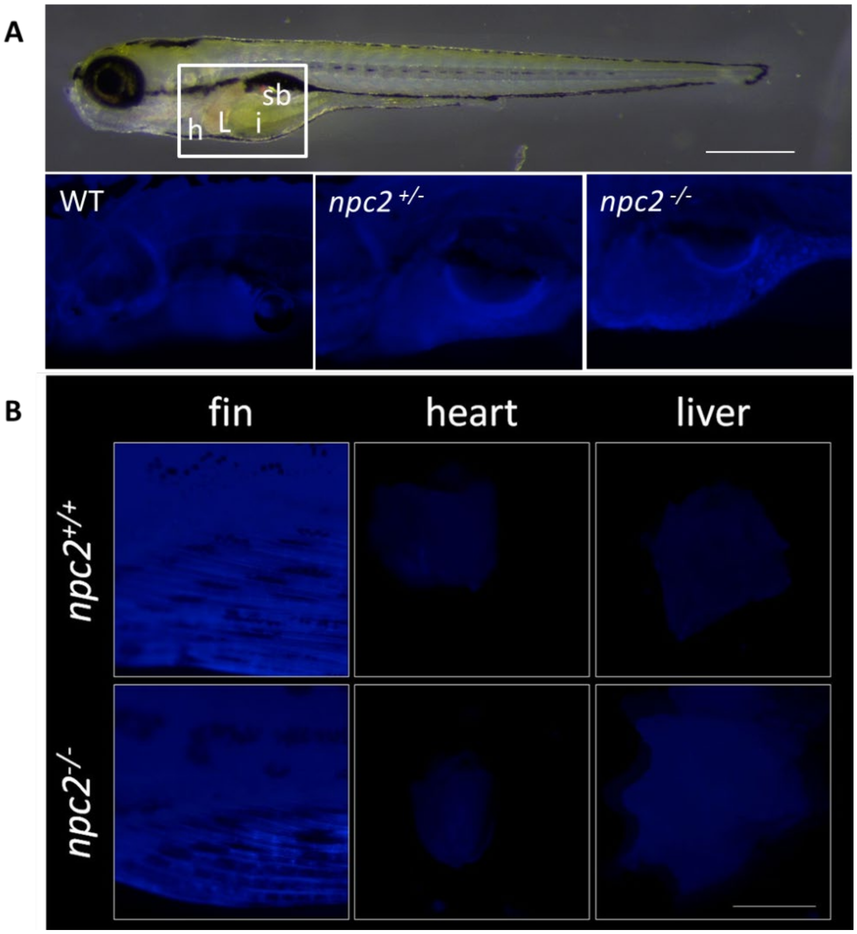


Figure S1. Un-esterified cholesterol accumulation in the *npc2* mutant. A, Bright puncta corresponding to accumulated cholesterol can be observed predominantly in the remaining of yolk and in the intestinal track in the *npc2*$^{-/-}$ larvae (lower panel). Top panel shows bright field image of the whole larvae. Rectangle indicates area shown in the lower panels. Lateral views: L- liver, sb- swim bladder, h- heart, i- intestines. B, Adult fish stained with filipin. Dissected heart (h), liver (L), and fins (f). Scale bar = 1 mm.

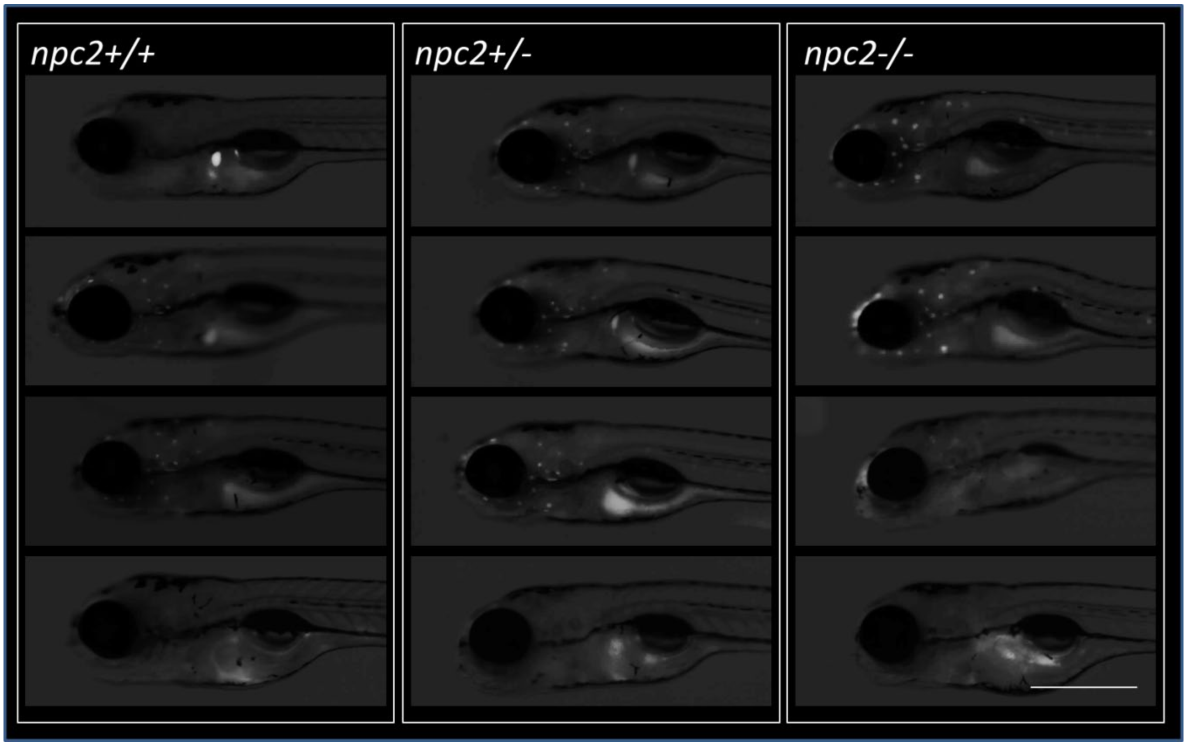


**Figure S2. LysoTracker red staining of the *npc2* mutant**. The fluorescent signal localized to neuromasts in wildtype and mutant fish at 5 dpf. High variability in staining intensity was found within and between wildtype, heterozygous, and homozygous *npc2* mutant fish, indicating that this staining cannot be used as a reliable method for identifying the homozygous *npc2* mutant. Lateral views of the anterior end are shown. Scale bar = 1 mm.

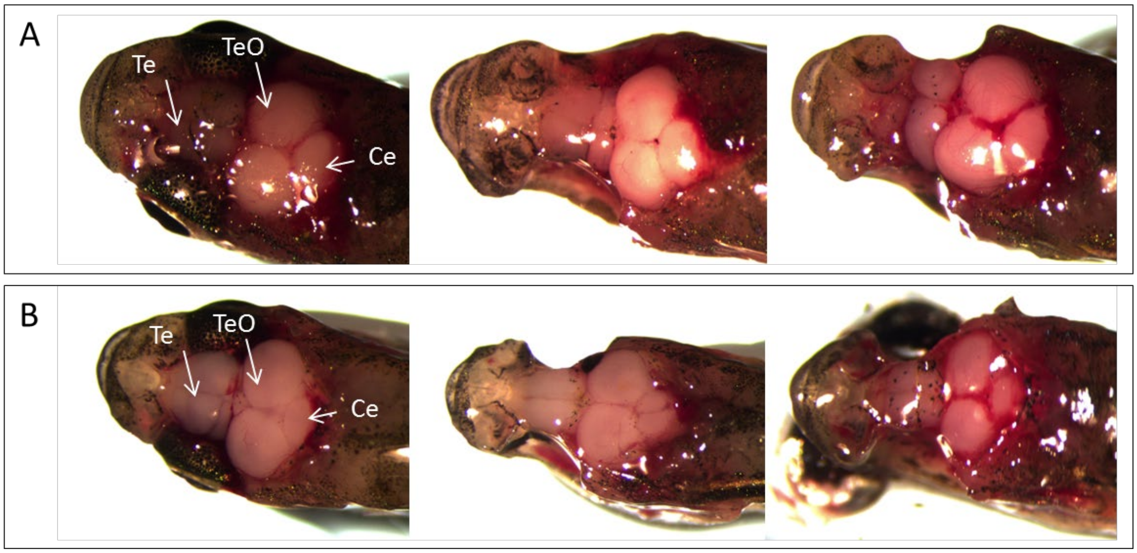


**Figure S3. Morphology of the *npc2*$^{-/-}$ brain.** Dorsal views of adult zebrafish brains are shown from three independent individuals, revealing a smaller cerebellum in *npc2* homozygous mutants. **A**. wildtype. **B**. *npc2*$^{-/-}$. Te, telencephalon; TeO, tectum opticum; Ce, cerebellum.

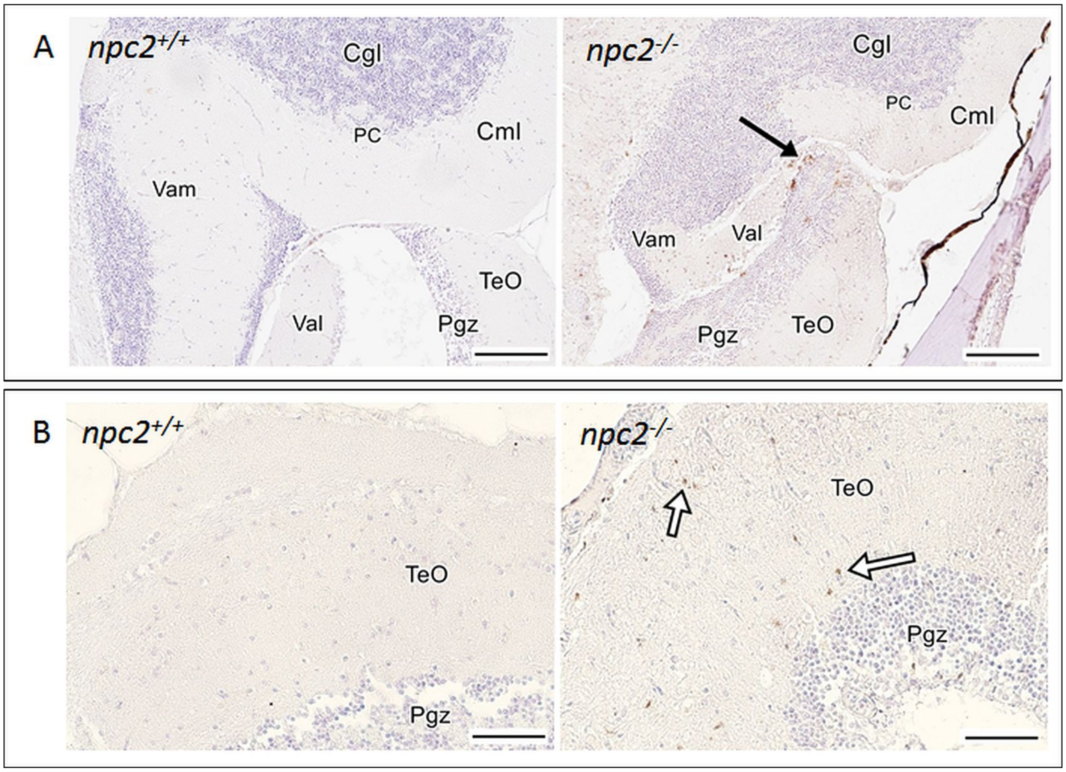


**Figure S4. Immunohistochemical localization of proliferating nuclear antigen and CD3 in central nervous system tissue in *npc2*$^{-/-}$ fish. A**. Proliferating cell nuclear antigen (PCNA), a marker of cellular proliferation, was detected in the caudal part of the torus longitudinalis of the optic tectum as a cluster of positive-stained cells (black arrow), whereas no PCNA-positive nuclei were observed in wildtype fish (*npc2*$^{+/+}$). **B**. Numerous, scattered, CD-3 positive cells (an immunohistochemical marker for T-cells) were present in the *npc2*$^{-/-}$ brain, especially in the optic tectum (black arrows), whereas no signal was detected in wildtype fish (*npc2*$^{+/+}$). Cgl, cerebellum granular layer, TeO, tectum opticum; Cml, cerebellum molecular layer; PC, Purkinje cell layer; Pgz, medial division of valvula cerebelli; Vam, periventricular gray zone of the optic tectum; Val, lateral division of the valvula cerebelli. Scale bar = 50 μm.

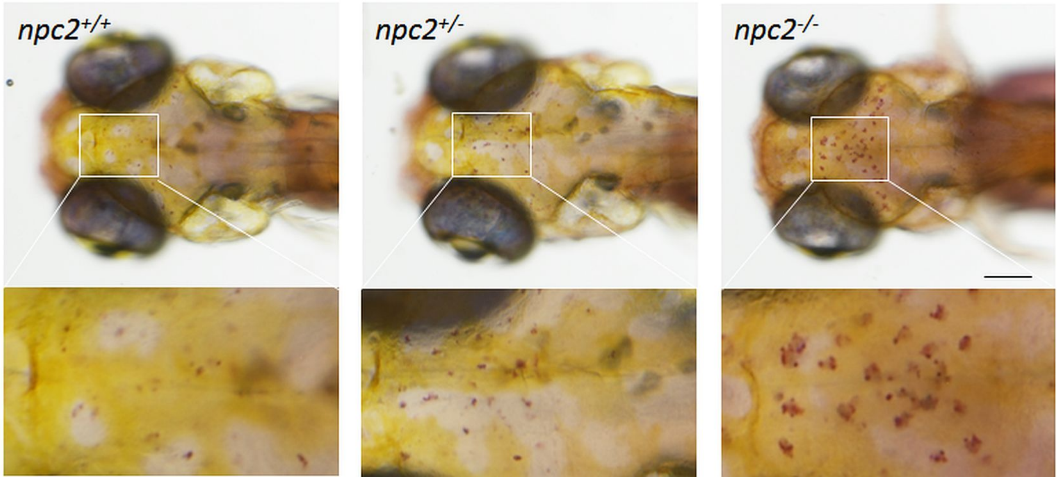


**Figure S5. Neutral red staining of the *npc2* mutant at 5 dpf**. Neutral red specifically labels microglia in the larval zebrafish brain. In contrast to WT and *npc2+/-*, microglia from *npc2*$^{-/-}$ fish are large, and have dark red aggregates within soma. Dorsal views of the zebrafish head are shown. Scale bar = 1 mm

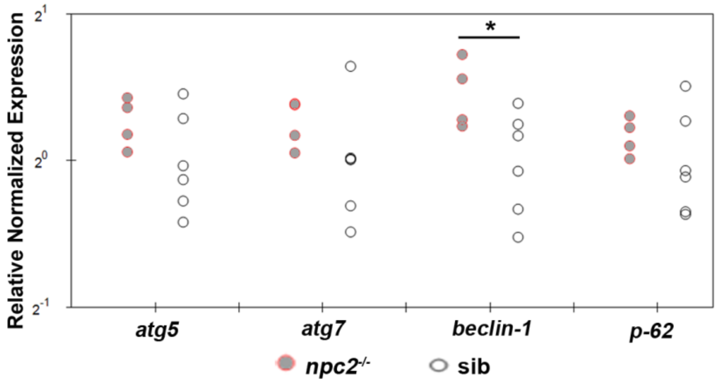


**Figure S6. Expression of the autophagy markers in *npc2*-deficient larvae and adult fish.** Scatter plots show the normalized expression of selected genes in 5 dpf larvae in $npc^{-/-}$ and wildtype zebrafish. Each circle corresponds to one zebrafish. The 18S ribosomal gene was used as a reference. At least three samples were analyzed. ***$p < 0.001$; **$p < 0.01$, *$p < 0.05$.
